# Sex differences in physiological correlates of affectively driven decision-making behavior in adult ADHD

**DOI:** 10.1186/s12888-024-06040-3

**Published:** 2024-09-05

**Authors:** Eva Halbe, Alina Sophie Heger, Fabian Kolf, Philippa Hüpen, Moritz Bergmann, Ben J. Harrison, Christopher G. Davey, Alexandra Philipsen, Silke Lux

**Affiliations:** 1 Department of Psychiatry and Psychotherapy, University of Bonn, Bonn, Germany; 2 Department of Psychiatry, Psychotherapy and Psychosomatics, Faculty of Medicine, RWTH Aachen University, Aachen, Germany; 3 JARA - Translational Brain Medicine, Aachen, Germany; 4 Department of Psychiatry, The University of Melbourne, Melbourne, VIC Australia

**Keywords:** Attention-deficit/hyperactivity disorder, Sex differences, Skin conductance response, Affective function, Risky decision-making behavior, Autonomic nervous system, Balloon analogue risk task, Physiological measurement

## Abstract

**Background:**

Sex differences in the symptomatology of adults with attention-deficit/hyperactivity disorder (ADHD) have often been overlooked when studying behavioral abnormalities. However, it is known that women exhibit considerably more stronger symptoms related to emotional competence than men. Since affective functions significantly influence the processing of risky decision-making and risk-engagement, we assume that risky behavior in ADHD is affected by sex differences. Therefore, we specifically investigated sex-specific effects on the interaction between emotionally induced changes in physiology and behavioral performance on a decision-making task.

**Methods:**

Skin conductance responses of twenty-nine adults with ADHD (*n* = 16 male; *n* = 13 female) and thirty-three adults in the control group (*n* = 14 male; *n* = 19 female) were recorded during the performance in a modified version of the Balloon Analogue Risk Task (BART). Additional questionnaires were used to reveal insights in the self-assessment of emotional competence, risk perception, and feedback sensitivity. Emotional arousal and decision-making behavior were analyzed using linear mixed-effects models.

**Results:**

Results showed different effects of sex on risk behaviors in controls and ADHD. In contrast to healthy controls, female adults with ADHD showed a significantly greater risk engagement in the BART compared to males with ADHD. This contrary sex relation was not observed in skin conductance responses and revealed a significantly different sex-dependent correlation of body response and behavioral task performance in ADHD. Comparisons with results from self-assessments furthermore indicate a reduced behavioral self-perception in women with ADHD, but not in men.

**Conclusion:**

In summary, we found an altered interaction between physiological activity and risky behavior in women with ADHD. Thus, the present study indicates a reduced sensitivity towards the own bodily responses in women with ADHD, which could consequently cause increased risky DM behavior in daily life. The current results suggest that more consideration needs to be given to sex-specific effects on physiological processes and behavior in adults with ADHD.

## Background

Attention-deficit/hyperactivity disorder (ADHD) is a multifaceted neurodevelopmental disorder that affects people across all ages worldwide [1]. Although ADHD is commonly considered a childhood disorder, however in up to 57% of diagnosed children, symptoms persist throughout life. Thus, research reveals that about 3% of adults worldwide live with ADHD [1–3]. Interestingly, while many studies report a sex gap in childhood diagnoses of ADHD, it has been found that women are often diagnosed later in life compared to men, indicating a converging sex ratio in adults with ADHD [4, 5]. Similarly, the symptomatology, which affects a wide range of neuropsychological functions has also been shown to undergo significant changes throughout life [6–8]. In this regard, the often seen typical symptoms of hypermotoric and impulsive behavior in childhood seem to decrease, whereas symptoms of internalizing difficulties, such as emotional dysregulation significantly increase [9, 10]. Notably, female patients experience more internalizing symptoms than males in both childhood and adulthood, whereas male patients exhibit more externalizing behavioral patterns. Although this characteristic of hypermotoric behavior also decreases in males as they transition into adulthood, the severity of these symptoms are less pronounced in women compared to men [4, 5, 11]. However, the relationship between internalizing and externalizing symptoms is far more complex than considering the two modes of functioning separately. For instance, underlying affective (internalizing) dysfunctions can also cause behavioral changes that apparently manifest as patterns of externalizing behavior [12, 13]. This article builds on our previous work, showing altered physiological activity as an indicator for challenges in affective functioning during a risky decision-making (DM) task in adults with ADHD [14]. In this context, we refer “affective functions” to underlying and mostly autonomous mechanisms, such as difficulties in unconscious emotional evaluation of events. The aim of the present study is to gain a deeper understanding of the sex differences in adult ADHD. Therefore, we investigate underlying mechanisms of internalizing processes of DM behavior, a common difficulty in adults with ADHD with particular focus on sex-specific differences.

There are only a few studies investigating sex differences in the symptomatology of individuals with ADHD. However, not only the differences in the representation of symptoms, but also the sex imbalance among individuals with ADHD provide reasons to examine sex-specific differences in ADHD more closely. While boys with ADHD often exhibit externalizing behavioral patterns that match the stereotypical clinical profile of the disorder, this condition in girls is frequently overlooked for a longer time. In this regard, the female symptomatology of ADHD often shows some overlap with other psychiatric conditions, primarily those related to mood disorders. This presents challenges for diagnosis and treatment management and can cause underdiagnoses due to diagnostic overshadowing [15–17]. In this regard, girls are significantly more likely than boys of the same age to receive an alternative diagnosis prior to their ADHD diagnosis. It has also been shown that girls are significantly more likely to be prescribed non-ADHD medications before their ADHD diagnosis compared to boys [18]. In this context, it can be assumed that the huge difference in sex ratio in childhood ADHD results from the high rate of more internalizing symptoms that are mainly prominent in girls and complicate the diagnosis of ADHD. Consequently, the imbalanced sex ratio in childhood ADHD could be a result of less salient behavior or misdiagnoses in girls. It could therefore be assumed that ADHD is more common in girls than the current prevalence suggests. In this context, it needs to be considered that an under-identification and under-treatment of girls with ADHD may have significant implications for mental health and education in later life [19]. Nevertheless, research on ADHD mainly consists of an imbalanced sex distribution and represents a clinical picture of ADHD biased towards male symptoms [11]. Therefore, further research is necessary to gain a better understanding of the clinical indicators of females with ADHD, as well as a deeper insight into affective functions and the resulting behavioral symptoms.

The linkage between emotional perception, processing, and behavior has been extensively investigated in previous studies. For instance, the dual pathway model illustrates that appropriate behavior results from the interplay between emotional-motivational functions and cognitive-analytical functions. Both functions are required to varying extents depending on the current event or decision that needs to be made [20]. For instance, affective functions play a particularly important role in quick and intuitive behaviors as they less rely on cognitive-analytical processes and more on underlying emotional-motivational mechanisms [20]. In this context, it is assumed that decisions towards risk-engagement are often intuitively processed and thus mainly driven by emotional-motivational processing and dependent on affective functions [21]. Risky DM is a frequently observed pattern of behavior in adults with ADHD, which not only impacts those affected but can also affect the health and safety of those around them [22]. These heightened risk-taking behaviors are particularly evident in situations involving risky driving, unsafe sexual practices, and pathological gambling [23–25]. Among the general population, a meta-analysis indicated increased risk-taking in men compared to women [26]. In laboratory settings, it has been shown that men perform better than women on a DM task, and furthermore, women were found to focus more on the valence of outcomes and less on the amount of rewards [27–29]. However, sex differences also seem to vary according to context and age level [26]. When risky DM behavior is considered in the context of deficits in affect, a psychiatric diagnosis has been shown to be a predictor of pathological gambling behavior in women but not in men [30]. The significance of increased risk behavior among individuals with ADHD in our society, along with the understanding that affective functions play a crucial role in the development of such behaviors, emphasizes the importance of investigating the relationship between emotion and risk behavior. Additionally, it raises the question of how the varying degrees of affective functioning between men and women with ADHD influence their decisions to take risks. To the best of our knowledge, no study so far investigated the impact of sex on affectively driven DM behavior in adults with ADHD. Thus, it is not clear how the significantly increased affective symptomatology in women with ADHD affect DM behavior. Given the importance of emotions and unconscious DM processes, it can be assumed that women with ADHD have more difficulties in this type of behavior.

Measuring skin conductance responses (SCR) has been proven to be a valuable method to detect affective states during performance on DM tasks. Moreover, changes in skin conductance represent unconscious processes before a decision is actually made [31–33]. Thus, physiological activities are quick responses of the body that are often described as intuition and are not actively controlled. According to the somatic marker hypothesis (SMH), anticipatory changes in SCR are considered as somatic marker that can guide DM without a sense of consciousness [34]. In context of risky DM, anticipatory increases in SCRs have also shown to be associated with decreased risk-engagement, so that physiological response may prevent engaging risky behavior [32, 33, 35, 36]. Thus, the detection of SCRs provides insights into affective reactions and can serve as an indicator of emotional processes. Regarding studies on affective functions in ADHD, it has been shown that SCRs in ADHD are altered during feedback processing as well as during anticipation of a risky decision [14, 37–40]. Moreover, these alterations were shown to be related to an altered risky DM behavior. Thus, it has been indicated that there might be a linkage between the affective symptoms and the behavioral patterns in individuals with ADHD. A study by Hermens et al., 2004 found that there are also sex differences in skin conductance activities in ADHD. Results indicated lower baseline activity in females compared to males, suggesting different psychophysiological processes in the two sexes [41]. However, females appear to be more variable in their SCRs and are more affected by SCR changes across the lifespan [42]. Nevertheless, a meta-analysis on physiological activities in ADHD showed that still a large number of studies increasingly focused on male outcomes while sex differences were largely neglected [43]. Furthermore, no study has yet investigated sex differences in risky DM and physiological changes, as indicators of affective functions, in adults with ADHD.

Overall, ADHD is characterized not only by a high heterogeneity in the clinical picture and a noticeable change in symptoms during lifetime, but also by sex differences regarding internal and external deficits in behavior [44]. Particularly, the impact of internal affective functions on behavior in adult ADHD is yet not fully understood. In our previous work, we found an altered interaction between physiological activity, representing underlying autonomous emotional processes, and performance on a modified version of the Balloon Analogue Risk Task (BART) [14]. With the present study, we aim to extend these findings by exploring the following research question: What impact does sex have on individuals with ADHD in relation to affectively driven DM behavior and the associated bodily function? Therefore, we replicated the study design from our previous work by using continuous recordings of SCRs during the performance of the modified BART to gain insights into the underlying affective functions related to DM behavior. The BART has been proven to capture real-life DM behavior, is suitable for trial-by-trial analysis, and shows moderate effect sizes when utilized in an ADHD cohort [6, 33, 45]. Furthermore, exploratory investigations using self-reports to measure self-perception of behavior and emotional competence were used to provide further understanding of sex-specific emotionally driven behavior in adult ADHD. In this context, we refer emotional competence to skills that are regulated by affective functions, such as deficits in emotional self-awareness, self-regulation, motivation, and social skills. Given the assumption that women with ADHD are more affected by internalizing and emotionally related symptoms, we suggest this characteristic is also reflected by increased difficulties in emotionally motivated behavior.

## Methods

### Participants

Thirty-three (*n* = 14 men; *n* = 19 women) healthy controls (HC) and twenty-nine (*n* = 16 men; *n* = 13 women) individuals with ADHD participated in the present study. All participants were between the age of 18 and 60 years, did not suffer from neurological diseases, were fluent in the German language and gave oral and written informed consent for participation. Demographic information of age, sex, educational level and medication intake were documented using a self-designed questionnaire. Participants with ADHD were asked to discontinue medication use for 24 h prior to their participation in the study. Psychiatric disorders in the HC group or comorbidities in the ADHD group were verified by a brief diagnostic interview (Mini-DIPS; [46]) and additional clinical questionnaires regarding depressive symptoms (Beck Depression Inventory-II; [47]) and borderline symptoms (Borderline Symptom List-95; [48]). ADHD symptoms were assessed by the Conners Adult Rating Scale (CAARS; [49]) and the validated short version of the Wender Utah Rating Scale (WURS-k; [50]). The raw scores of the CAARS and the WURS-k were transformed into t-scores to identify clinically relevant values regarding ADHD symptoms (t-scores > 65 are considered clinically significant). Participants with deviations from the normal range values (± 1 SD) in the clinical questionnaires or incomplete data (missing items in questionnaires or incomplete SCR recordings) were excluded from further analyses. Participants with ADHD were recruited from the outpatient clinic of the Department of Psychiatry and Psychotherapy at the University Hospital Bonn, as well as from surrounding psychotherapeutic private practices. This clinical recruitment process ensured that all participants with ADHD had a confirmed diagnosis according to the DSM-5 criteria [51]. HCs were recruited via public advertisement on the Internet and flyers. The study was approved by the Ethics Committee of the Medical Faculty of the University of Bonn (122/21).

### Self-reported questionnaires

#### Emotional competence

To assess different traits in the emotional competence, we used the questionnaire „Emotionale Kompetenz Fragebogen, (EKF)“ [52]. This questionnaire is designed to evaluate self-assessment within four categories (recognizing own feelings, recognizing emotions of others, regulation and control of own feelings and emotional expressivity) and a resulting total score describing the emotional competence. The EKF consists of 62 items that should be rated using a Likert-Scale (1-5). For further analyses, a sum score was used. The assumed factor structure has been confirmed by Rindermann (2009), showing high reliability across the scales (average Cronbach’s α = 0.91) and moderate stability over a year (average *r* = 0.69).

#### Risk perception

To assess self-rating of risk attitude and tolerance, we used the questionnaire „Domain Specific Risk Taking “ (DOSPERT; [53]). The questionnaire is designed to assess the attitude towards risk and consists of 40 items describing daily situations. Using a Likert-Scale (1–5), each item should be rated in three categories: the likelihood of engaging in a certain risk behavior (prob), the perception of risk (risk), and the expected utility of a specific situation (ben). In addition, each item is assigned to one of the five following subdomains to assess risk behavior in different areas of life: Investment, Gambling, Health, Recreational, Ethical and Social. For further analyses, sum scores for each subdomain were used. The German version of the DOSPERT was validated in a study with 532 participants, showing moderate reliability overall. The highest reliability was observed in the gambling dimension (Cronbach’s α = 0.82 for prob, Cronbach’s α = 0.85 for risk, Cronbach’s α = 0.83 for ben), while the lowest reliability was in the social dimension (Cronbach’s α = 0.51 for prob, Cronbach’s α = 0.63 for risk, Cronbach’s α = 0.56 for ben).

#### Feedback sensitivity

To assess self-rating of aversive and approach behavior, we used a German version of the questionnaire „Sensitivity to Punishment and Sensitivity to Reward Questionnaire “ (SPSRQ; [54]). This questionnaire is based on the two motivational systems (Behavioral Inhibition System and Behavioral Activation System) that can control certain behavior. To assess which of the two systems are more likely to control behavior, the questionnaire consists of 48 items that are assigned to either sensitivity towards punishment or sensitivity towards reward. The questions are dichotomous, with each "yes" being scored with one point and each "no" with zero points. The ratio of the total sums indicates which category is predominant. The questionnaire demonstrates acceptable to good internal consistency being Cronbach’s α = 0.83 for sensitivity to punishment and Cronbach’s α = 0.77 for sensitivity to reward.

#### DM paradigm

In order to assess risky DM behavior, we used a modified version of the Balloon Analogue Risk Task (BART). The modified BART was designed with Presentation® software of neurobehavioral systems (www.neuro bs.com). See Henn et al. 2023 for detailed description of the paradigm [55]. Compared to the original version the modified BART enables continuous measurements of affective driven DM behavior. Due to the new design of a dynamically and automatically inflating balloon, participants were asked to make decisions based on intuition, which is closely linked to emotional and motivational processes. Therefore, the task consists of 60 consecutive trials and aims to collect virtual money. Each trial starts with displaying the potential amount of reward (1500 ms) that can be gained and is additionally highlighted by colors whether it is a high (yellow; *n* = 30 trials) or low (white; *n* = 30 trials) reward condition. Afterwards, an automatically dynamically inflating balloon appears on the screen. The participants are informed that with increasing size of the balloon the amount of money is also increasing. However, the increasing size of balloon also coincides with greater risk that the balloon explodes, and if so the already collected money of the current trial will be lost. Thus, in order to save the money and prevent the loss due to an explosion, the participant can press a response button during the period of inflation. The duration of the inflation period in every trial is 5000 ms and thus does not allow the participant to visually perceive the explosion of the balloon. Following this period, a fixation cross appears on the screen (250 ms) followed by a feedback display (2500 ms), that either inform about success and money cash-out or failure and money loss. The monetary amount of the current trial as well as the sum of total amount are displayed with feedback in each trial. Risk engagement and more disadvantageous DM behavior are measured by the response time (RT); longer RTs indicate higher levels of risk-taking behavior [51, 56, 57].

### Skin conductance

#### Apparatus

The Biopac 150 system (Biopac Systems, Inc.) was used for recording skin conductance. Via the wireless PPG/EDA BioNomadix Transmitter recordings were transferred to the software AcqKnowledge on a recording computer that is used for data acquisition and analysis. Simultaneously, the recording computer is synchronized with the experimental computer presenting the paradigm, via digital input ports to receive event trigger of the behavioral performance in the task. Skin conductance is acquired by disposable snap (Ag–AgCl) electrodes (11 mm diameter) from the palm of the non-dominant hand.

#### Procedure

Two electrodes were prepared with a 0.5% saline paste in a neutral base (0.05 molar NaCl) and were attached to the thenar and hypothenar eminence of the participants’ hand. Recordings were continuously taken during the performance in the BART at 5,000 Hz and a direct current excitation of 0.5 V. Before preprocessing, relevant event trigger of response time, reward condition, and feedback display were extracted from the recordings while using a transition latency of 2 ms and low pass filtering of 1 Hz. For further processing regarding identification of artefacts and applying downsampling to 20 Hz, Ledalab toolbox (V.3.4.8) of Matlab was used. Final preprocessed data was analyzed using a continuous decomposition analysis (CDA) to reveal relevant phasic SCR that can be related to an event trigger from the paradigm. Therefore, short peaks with a minimum amplitude criterion of 0.05 µS within fixed response windows were identified as affective driven changes in physiological activity. For anticipatory SCRs prior to a decision a response window of 1 to 5 s after condition display was used. For reactive SCRs regarding feedback processing a response window of 1 to 2.5 s after feedback display was used.

### Statistical analyses

Group comparisons regarding results in self-assessment of emotional competence were performed using a multifactorial Analysis of Variance (ANOVA) with group (ADHD; HC) and sex (male; female) as independent variables. Analyses of self-assessment in the three subdomains of the DOSPERT (prob; risk; ben) and the two subdomains of the SPSRQ (SP; SR) were performed using multifactorial multivariate Analyses of Variance (MANOVA) with group (ADHD; HC) and sex (male; female) as independent variables. Assumptions of homogeneity of variances were tested using Levene Test. Post hoc group comparisons were calculated using Tukey HSD test. Analyses of the behavioral data and physiological measures were performed using linear effects model containing interaction terms of the fixed effects and random intercepts for participants and trials. In order to analyze group differences related on sex, both variables were included as fixed factor in every model. Response time (RT), anticipatory SCRs and reactive SCRs were each included as dependent variables in three separate models. As additional fixed effect, feedback (gain; loss) was included in the model investigating reactive changes in SCRs, whereas reward condition (high; low) was included in models investigating behavior, anticipatory SCRs and their relation. All analyses were performed using R [58]. As the reward condition did not reveal sufficient effects in the models of our previous study, it was excluded from the models and for further interpretation of results. Model effect sizes were calculated. Therefore, the total explanatory power is described by the conditional R^2^, whereas the part related power to fixed effect is described by the marginal R^2^. In terms of conditional R^2^, values < 0.1, between 0.1 and 0.3, and > 0.5 were considered to be small, medium and large effect sizes. For marginal R^2^, values < 0.02, between 0.02 and 0.13, and > 0.26 were considered to be small, medium and large effect sizes. Considering the small-to-medium effect sizes using the BART as a DM task, an a priori power analysis accounting for two within-subject factors and a repeated measures design (60 trials per participant) indicates that 58 participants are sufficient for a robust examination of our hypotheses [45, 59].

## Results

### Demographics

The group of participants with ADHD were comparable to the group of HC on sex ratio (χ^2^(1) = 1.004, *p* = 0.316, ϕ = 0.127). There were no significant group differences with regard to age and years of education (see Table 1). Screening of depressive and borderline symptoms by clinical questionnaires did not reveal any abnormalities in either group. Thus, no subject was excluded for further data analyses. The following types of ADHD-related medications were documented: Elvanse, Medikinet, and Ritalin. All participants ceased medication intake prior to their participation in the study.

**Table 1:** Demographic characteristics and clinical ADHD symptoms

*Parameter*	Median				Mann–Whitney-U-Test	
	ADHD		HC			
	males^**1**^ (*n* = 16)	females^**2**^ (*n* = 13)	males^**3**^ (*n* = 14)	females^**4**^ (*n* = 19)	within-group comparison	Between-group comparison
**Age (years)**	31.50	27.00	30.00	26.00	^1=2^ U = 87.5, *p* = .48 ^2=3^ U = 88.5, *p* = .11	^1=3^ U = 110, *p* = .95 ^2=4^ U = 115.5, *p* = .76
**Education (years)**	16.50	16.25	18.00	16.50	^1=2^ U = 95.5, *p* = .98 ^2=3^ U = 76.5, *p* = .11	^1=3^ U = 74, *p* = 0.2 ^2=4^ U = 108, *p* = 1
**CAARS**^**a)**^_(**Hyperactivity)**_	23.50	24.00	11.00	9.00	^1=2^ U = 93.5, *p* = .65 ^2=3^ U = 95, *p* = .17	^1> 3^ U = 195, *p* < .001 ^2 > 4^ U = 220, *p* < .001
**CAARS**_**(Inattention)**_	25.50	22.00	12.50	8.00	^1=2^ U = 88.5, *p* = .5 ^2=3^ U = 100.5, *p* = .24	^1 > 3^ U = 194.5, *p* < .001 ^2 > 4^ U = 230, *p* < .001
**CAARS**_**(Impulsivity)**_	19.00	20.00	6.00	6.00	^1=2^ U = 98, *p* = .81 ^2=3^ U = 108, *p* = .38	^1 > 3^ U = 200, *p* < .001 ^2 > 4^ U = 233, *p* < .001
**CAARS**_**(Self-conception)**_	13.00	11.00	5.50	4.00	^1=2^ U = 98.5, *p* = .81 ^2=3^ U = 118.5, *p* = .6	^1 > 3^ U = 172, *p* < .001 ^2 > 4^ U = 210.5, *p* = .012
**WURS-k**^**b)**^	15.50	40.00	15.50	11.00	^1=2^ U = 96.5, *p* = .75 ^2=3^ U = 93, *p* = 0.15	^1 > 3^ U = 204, *p* < .001 ^2 > 4^ U = 240, *p* < .001

Sign. alpha level at *p* < 0.05

^a^Conners Adult Rating Scale (raw scores)

^b^Wender Utah Rating Scale (raw scores)

^*^1 = ADHD males; 2 = ADHD females; 3 = HC males, 4 = HC females

### Self-assessment

The ANOVA for the total score of emotional competence (EKF) revealed no significant main effect of sex (F_(1,58)_ = 1.67, *p* = 0.202), while there was a significant effect of group (F_(1,58)_ = 18.33, *p* < 0.001). Results indicated higher emotional competence in HC (M = 82.29 ± 12.17) compared to ADHD (M = 66.81 ± 14.97). The MANOVA for total scores of the DOSPERT revealed no significant effect of group in all three subdomains (prob: F_(1,58)_ = 1.74, *p* = 0.19; risk: F_(1,58)_ = 0.02, *p* = 0.88; ben: F_(1,58)_ = 0.29, *p* = 0.59). However, there was a significant effect of sex in the subdomain probability of risk engagement (prob: F_(1,58)_ = 6.28, *p* = 0.015), whereas risk estimation (risk: (F_(1,58)_ = 1.19, *p* = 0.28) and evaluation of benefit (ben: F_(1,58)_ = 0.041, *p* = 0.841) did not significantly differ between males and females (see Table 2). Results showed significant higher probability of risk engagement (prob) in males (M = 97.4 ± 20.34) compared to females (M = 85.72 ± 18.02). The MANOVA for total scores of sensitivity to reward (SR) and sensitivity to punishment (SP) revealed no significant effect of sex (SR: F_(1,58)_ = 1.97, *p* = 0.17; SP: F_(1,58)_ = 0.06, *p* = 0.81) and no significant effect of group (SR: F_(1,58)_ = 0.01, *p* = 0.92; SP: F_(1,58)_ = 3.05, *p* = 0.09).

**Table 2:** Group comparison of total scores of self-reported questionnaires

*Parameter*	Mean (SD)				Analysis of Variance					
	ADHD		HC		Main effect				Interaction effect	
	males^**1**^ (*n* = 16)	females^**2**^ (*n* = 13)	males^**3**^ (*n* = 14)	females^**4**^ (*n* = 19)	Group		Sex		Group x Sex	
					F	*p*	F	*p*	F	*p*
**EKF**^a^	64.3 (16.9)	69.9 (12.2)	80.4 (12.7)	83.7 (11.9)	F_(1,58)_ = 18.33	< .001	F_(1,58)_ = 1.67	.20	F_(3,58)_ = 7.26	< .001
**DOSPERT**^b^_**(prob)**_	93 (16.12)	83.6 (18.1)	102.4 (23.9)	87.2 (18.3)	F_(1,58)_ = 1.74	.19	F_(1,58)_ = 6.28	.02	F_(3,58)_ = 2.61	.06
**DOSPERT **_**(risk)**_	132.6 (25.9)	135.3 (25.2)	127.8 (15.4)	138.2 (24.3)	F_(1,58)_ = 0.02	.88	F_(1,58)_ = 1.19	.28	F_(3,58)_ = 0.57	.64
**DOSPERT **_**(ben)**_	99.9 (18.1)	99.9 (24.2)	98.1 (17)	95.9 (22.8)	F_(1,58)_ = 0.29	.59	F_(1,58)_ = 0.041	.84	F_(3,58)_ = 0.14	.94
**SP**^c^	10.6 (5.9)	11.8 (7)	8.9 (4.9)	8.4 (4.9)	F_(1,58)_ = 3.05	.09	F_(1,58)_ = 0.06	.81	F_(3,58)_ = 1.12	.35
**SR**^d^	10 (3.25)	9.54 (4.7)	10.86 (4.24)	8.47 (3.71)	F_(1,58)_ = 0.01	.92	F_(1,58)_ = 1.97	.17	F_(3,58)_ = 1.04	.38

Sign. alpha level at *p* < 0.05

^a^Emotionale-Kompetenz-Fragebogen (min–max range: 19 – 117)

^b^Domain Specific Risk Taking (probability of risk engagement (prob); risk estimation (risk); estimation of benefit (ben); min–max range: 40–200)

^c^Sensitivity to punishment (min–max range: (0 – 24)

^d^Sensitivity to reward (min–max range: 0 – 24)

^*^1 = ADHD males; 2 = ADHD females; 3 = HC males, 4 = HC females

#### DM behavior

**Fig. 1:**
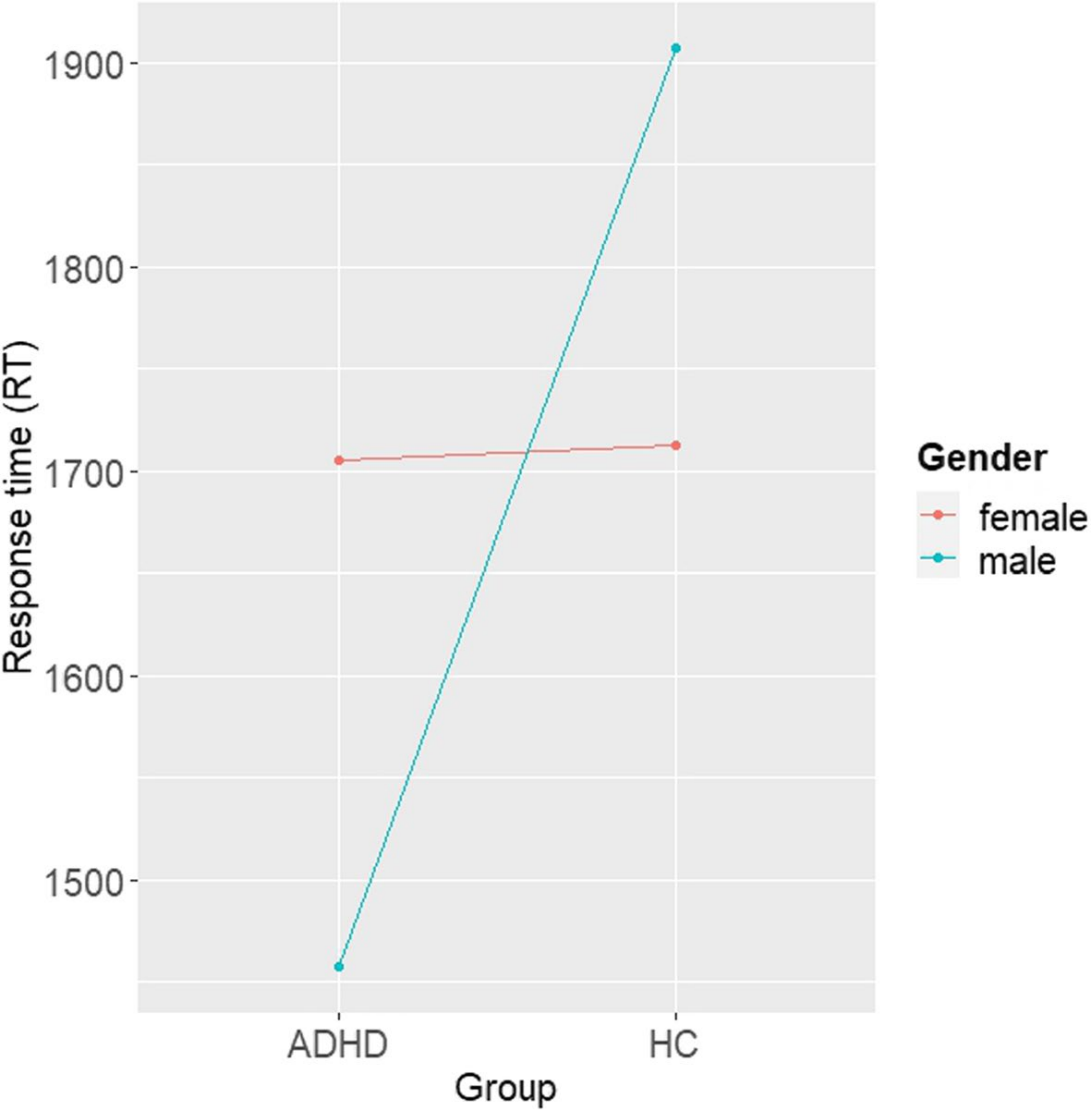
Interaction effect of response time (RT). Representing group differences (ADHD, HC) for mean RT in females (red) and males (blue)

The first linear effects model investigated group differences in risky DM on basis of the RT in the BART according to sex (see Fig. 1, Table 3). The model revealed a significant main effect of group (ß = -116.64, SE = 54.59, t = -2.14, *p* = 0.033) and sex (ß = -498.39, SE = 60.37, t = -8.26, *p* < 0.001) with a conditional effect size of R^2^ = 0.54 and a marginal effect size of R^2^ = 0.03. Furthermore, there was a significant interaction effect of group and sex (ß = 610.44, SE = 98.83, t = 6.18, *p* < 0.001). Post hoc analyses indicated significant higher RT in females compared to males in the group of ADHD (M_ADHD,female-male_ = 498, SE = 60.4, *p* < 0.001), whereas RT was not significantly different between sex in the group of HC (M_HC,female-male_ = -112, SE = 62.6, *p* = 0.073).

**Table 3:** Parameter estimates from the linear mixed effects model analyses

**Model**		**b**	**SE**	**t**	**CI 95%**	***p***
**RT**^a^	Group	-116.64	54.59	-2.14	[-223.67, -9.62]	0.033
	Sex	-498.39	60.37	-8.26	[-616.75, -380.03]	< 0.001
	Group x Sex	610.44	98.83	6.18	[416.68, 804.21]	< 0.001
**rSCR**^b^	Group	0.01	0.09	0.13	[-0.16, 0.18]	0.897
	Feedback	0.03	0.07	0.42	[-0.11, 0.17]	0.678
	Sex	0.44	0.1	4.58	[0.25, 0.62]	< 0.001
	Group x Feedback	-0.05	0.09	-0.55	[-0.24, 0.13]	0.582
	Group x Sex	-0.11	0.15	-0.76	[-0.4, 0.18]	0.446
	Sex x Feedback	-0.29	0.1	-2.97	[-0.48, -0.1]	0.003
	Group x Feedback x Sex	0.34	0.14	2.48	[0.07, 0.61]	0.013
**aSCR**^c^	Group	-0.16	0.07	-2.34	[-0.3, -0.03]	0.02
	Sex	0.08	0.08	1.05	[-0.07, 0.23]	0.293
	Group x Sex	0.32	0.12	2.63	[0.08, 0.57]	0.009
**aSCR x RT**	aSCR	-129.16	31.27	-4.13	[-190.46, -67.86]	< 0.001
	Group	-184.21	58.12	-3.17	[298.17, -70.25]	0.002
	Sex	-538.23	64.98	-8.28	[-665.63, -410.83]	< 0.001
	aSCR x Group	195.41	43.38	4.5	[110.36, 280.47]	< 0.001
	aSCR x Sex	144.06	41.53	3.47	[62.64, 225.48]	< 0.001
	Group x Sex	724.48	103.07	7.03	[522.39, 926.57]	< 0.001
	aSCR x Group x Sex	-202.08	54.77	-3.69	[-309.46, -94.69]	< 0.001

Sign. alpha level at *p* < 0.05

Linear mixed-effects model with group (ADHD, HC) as fixed factor in every model. Sex (female, male) was additionally included as fixed factor in every model. Investigating risky behavior based on the RT, a model was fitted with RT as dependent variable. Investigating physiological response for feedback, a model was fitted including feedback as additional fixed effect and reactive SCR as dependent variable. Investigating physiological response anticipating a DM, a model was fitted with anticipatory SCR as dependent variable. Investigating the interaction of physiology and behavior, a model was fitted including anticipatory SCR as additional fixed effect and RT as dependent variable

^a^Response time

^b^Reactive skin conductance response

^c^Anticipatory skin conductance response

#### Physiological activity

The second linear effects model investigated group and sex differences in arousal during feedback display on basis of the reactive SCRs and according to the feedback (see Table 3). The model revealed a significant main effect of sex (ß = 0.44, SE = 0.1, t = 4.58, *p* < 0.001) with a conditional effect size of R^2^ = 0.6 and a marginal effect size of R^2^ = 0.01. Furthermore, there was a significant interaction effect of group, sex, and feedback (ß = 0.34, SE = 0.14, t = 2.48, *p* = 0.013). Post hoc analyses indicated significant higher reactive SCRs during loss feedback in males (M_male,loss-gain_ = 0.12, SE = 0.05, *p* = 0.019) and with a more prominent effect in ADHD compared to HC (M_ADHD,loss-gain_ = 0.15, SE = 0.05, *p* = 0.019).

The third linear effects model investigated group and sex differences in arousal prior to a DM on basis of the anticipatory SCRs (see Table 3). The model revealed a significant main effect of group (ß = -0.16, SE = 0.07, t = -2.34, p = 0.02) with a conditional effect size of R^2^ = 0.45 and a marginal effect size of R^2^ = 0.01. Furthermore, there was a significant interaction effect of group and sex (ß = 0.32, SE = 0.12, t = 2.63, *p* = 0.009). Post hoc analyses indicated significant higher anticipatory SCRs in males in the group of HC (M_HC,female-male_ = -0.4, SE = 0.08, *p* < 0.001) and no sex differences in the group of ADHD.

#### Behavior and physiology

**Fig. 2:**
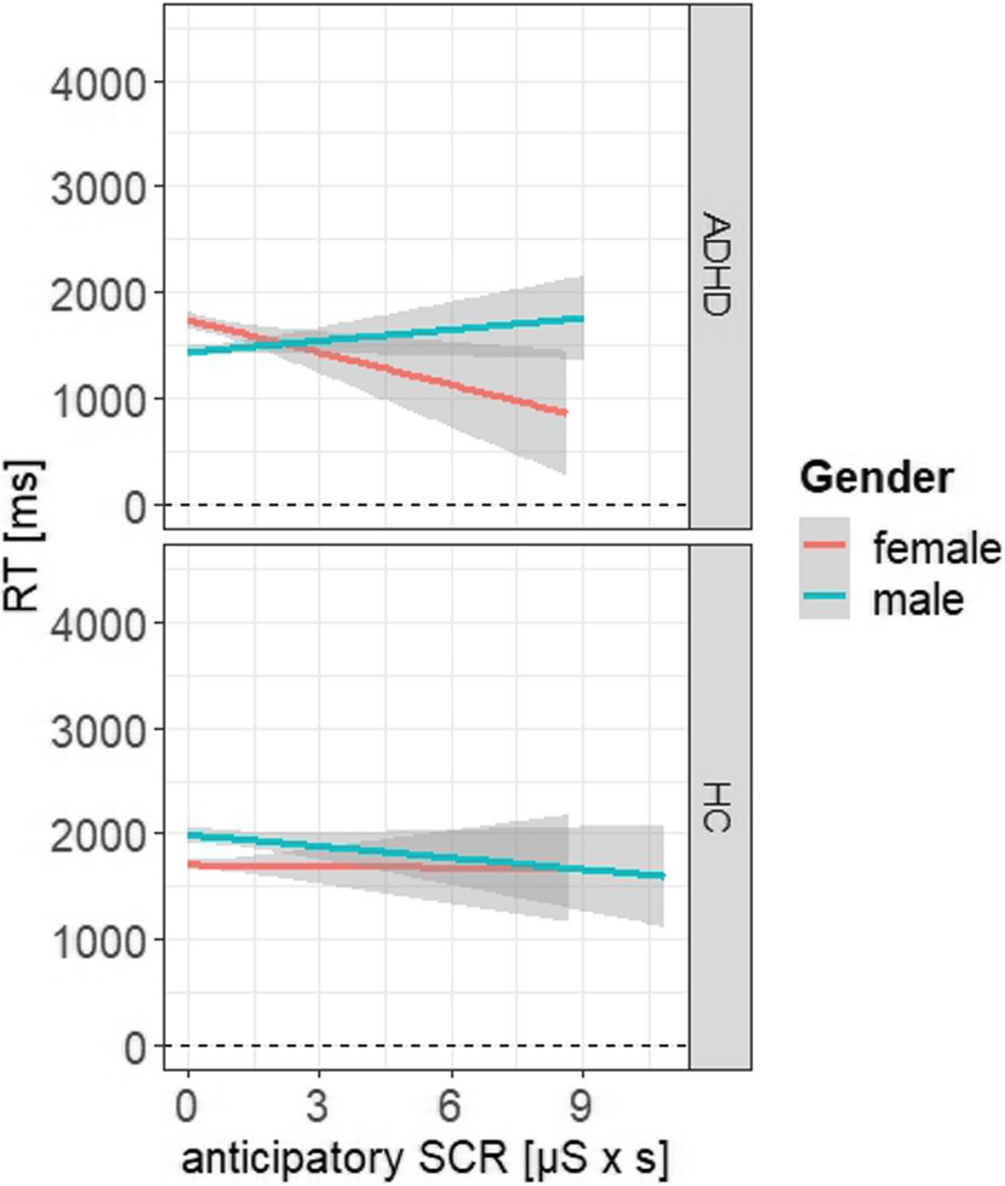
Interaction effect of anticipatory SCR and response time (RT). Representing the simple slopes for the interaction at the factor variables group (ADHD, HC) dependent on sex (male, female)

The fourth linear effects model investigated the impact of anticipatory SCRs on the RTs and the differences in groups and sex (see Fig. 2, Table 3). The model revealed again a main effect of group and sex, but also for anticipatory SCR (ß = -129.16, SE = 31.27, t = -4.13, *p* < 0.001), with a conditional effect size of R^2^ = 0.42 and a marginal effect size of R^2^ = 0.04. Furthermore, the model revealed a significant interaction of anticipatory SCR and group (ß = 195.41, SE = 43.38, t = 4.51, *p* < 0.001) and anticipatory SCR and sex (ß = 144.06, SE = 41.53, t = 3.47, *p* < 0.001). There was also a significant three-way interaction effect of anticipatory SCR, sex and group (ß = -202.08, SE = 54.77, t = -3.69, *p* < 0.001). Post hoc analyses indicated decreasing RT in HC when anticipatory SCR is high in males, whereas there is no SCR related change in RT in females (M_HC,female-male_ = -156, SE = 64.9, *p* = 0.016). An opposite effect is indicated in the group of ADHD, with increasing RT when anticipatory SCR is high in males and decreasing RT when anticipatory SCR is high in females (M_ADHD,female-male_ = 464, SE = 61.4, *p* < 0.001).

## Discussion

The present study investigated sex differences in affective-motivational driven behavior related to physiological processes in adults with ADHD compared to HC. Results were furthermore examined with respect to the self-assessment in emotional competence, risk perception and feedback sensitivity. The results reflect an altered relation between affective functions, indicated by the physiological activity, and the subsequent DM behavior guided by this body response, in women with ADHD compared to men with ADHD.

Using questionnaires, we revealed insight into self-perception of risky DM behavior in ADHD. Regarding self-assessment of emotional competence, the results showed significant group differences indicating a reduced ability for recognizing and treating own/other feelings in participants with ADHD. These results align with the frequently observed and reported deficits in affective functioning of people with ADHD, highlighting the underlying mechanisms involved in abilities related to emotional competence [60, 61]. However, we could not identify a sex difference in the self-assessment of emotional competence on the basis of the questionnaire. Since the assessed emotional competence represents conscious skills in dealing with emotions, it can be assumed that it is less about conscious handling and more about underlying affective functions that may cause sex differences in adult ADHD. Furthermore, no significant effects were observed regarding the often shown altered sensitivity to feedback in ADHD. In this regard, it is important to consider that previous study results have indicated that self-reports are less strongly associated with the competencies of ADHD compared to reports from others [62]. On the other hand, significantly higher scores on self-assessed probability of risk engagement (prob) were found in males, whereas this sex effect did not differ between both groups. As a previous study has indicated, sex-specific symptoms may appear differently on subjective and objective measures [63]. In this context, reports from teachers showed greater psychiatric internalizing deficits in boys, whereas most clinical measures identified more severe affective impairment in females [64]. Another study also confirms these inhomogeneities and emphasizes the need for complementary objective measures in screening of ADHD symptoms [65]. Consequently, the results of the current study do not indicate strong evidence for sex differences in the self-assessment. Nevertheless, as ratings in questionnaires represent a subjective and consciously made evaluation, the results reveal an interesting insight in the self-perception of feelings and behavior in adult ADHD. Thus, the current results of the questionnaires used indicate that deficits regarding affective functions seem to be perceived, whereas risky behavior does not appear to be deliberately engaged. This potential lack of awareness regarding risk estimation in individuals with ADHD aligns with a previous study, which indicated that risk perception did not mediate the association between ADHD symptoms and risk-taking behavior [66]. Since emotional competence is also based on completely unconscious processes of affective functions, it can be inferred from the results that underlying mechanisms of emotion processing, which are not consciously perceived, are related to behavioral problems in adults with ADHD.

Investigating to what extend risky DM is objectively reflected, the performance in the modified BART was analyzed with respect to sex. The results indicate that behavioral task performance considerably depends on sex. Thus, female participants showed significantly greater risky DM behavior, indicated by the RT than male participants with ADHD, whereas no significant sex-differences were found in the group of HC. With these results, the present study is the first to demonstrate that sex differences in affective driven risky DM behavior exist in adult ADHD. Since the modified version of the BART used in the current study is intended to require unconscious, intuitive DM behavior with help of undeliberate affective functions, the hypothesis can be confirmed that in particular women with ADHD are affected by deficits in emotional-driven DM. However, it needs to be considered that both women and men with ADHD did not exhibit significantly greater risky DM behavior compared to HC. Moreover, it seems contradictory that the least risky DM behavior was found in the group of male participants with ADHD. A possible interpretation that is often used to explain the reduced applicability of daily life behaviors to laboratory settings, is that individuals with ADHD have learned behavioral strategies to cope with their instincts [7]. Taken together, however, it appears that exhibited behavior alone is not sufficient to explain the effect of sex on the symptomatology of adults with ADHD.

The further exploration of the underlying affective processes measured by the skin conductance in response to feedback stimuli in the task and the undeliberate anticipation of DM, indicated overall higher responsiveness of the autonomic nervous system in males than in females in both ADHD and HC. These sex-related differences were also reported in previous studies that for instance indicate attenuated sympatho-adrenal activation in women [67]. The current study showed that elicited effects in the reactive SCRs were more prominent during negative feedback compared to positive feedback with a greater effect in the ADHD group. Such a blunted relation of positive emotional experience and sympathetic response was also reviewed by a recent meta-analysis [68]. Furthermore, greater physiological responses to losses than to gains have also successfully shown in studies on DM behavior [56, 69]. Although the self-assessment results in the current study did not indicate increased sensitivity to punishment, there is some evidence in the literature that individuals with ADHD perceive emotional stimuli as more arousing than HC and are particularly distracted by stimuli with negative valence [57, 70–72]. Regarding physiological activity during anticipation of a DM, men also exhibited higher amplitudes than women in both the ADHD group and the control group. However, the sex differences in anticipatory SCRs were only significant between women and men with ADHD, but not between the sexes in the control group. Consequently, the current results support the hypothesis that increasingly women with ADHD have deficits in the autonomic response towards external stimuli that might cause affective dysfunctions.

To further explore the extent to which altered sex-specific physiological activity modifies behavioral performance, anticipatory SCRs were also analyzed in relation to subsequent behavior. Results indicated a negative association of anticipatory SCR and RT in male HC and female ADHD, whereas the correlation was shown to be positive in male ADHD and was not present in female HC. The relationship between DM and anticipatory changes in the physiological activity has been proven in previous studies in context of conditioned learning of reward and punishment contingencies but also in laboratory settings where no learning was required [73–75]. However, this interconnection between the preceding physiological response and the task performance is difficult to generalize for both types of DM (affective-driven and cognitive-driven). While deliberate risk-taking (choosing inflation versus cashout) in the original BART is associated with a positive correlation of risky behavior and anticipatory SCR, results in the modified version of BART indicate a negative correlation of disadvantageous behavior and physiological activity. Here, less risky performance, as seen in male ADHD and female HC, could be traced back to an intrinsic warning signal of the autonomic nervous system. Whereas in the original BART the behavior shown is usually correlated with the general individual body function, in the current study anticipatory activity is associated with the subsequent behavior in each trial [55, 76, 77]. Thus, the results reflect more the temporal sequence of physiology and behavior. Therefore, this relationship suggests a certain sensitivity to bodily response, implying that individuals with a positive correlation of anticipatory SCR and RT are more sensitive to their body functions and thus less risky. The present results indicate a reduced sensitivity towards the own bodily responses in women with ADHD, which could consequently cause increased risky DM behavior. Similarly, the negative correlation among the men in the control group also indicates a reduced sensitivity to their own bodily responses, which is in line with the existing literature. For instance, a study investigating interoceptive awareness suggested that men perceive bodily sensations less frequently and recognize the relationship between bodily sensations and emotional states less effectively compared to women [78]. Interestingly, neither a positive nor a negative correlation was found among the women in the control group, which could possibly be related to a kind of indifference to the emerging risk. Thus, it can be suggested that women in the control group were less attracted to gambling (potential winnings), so no correlation with emotional arousal was observed.

However, some limitations must be taken into account considering results of physiological parameters. In this context, individual and environmental influences on the measurements can occur, which may introduce artifacts and biases in data collection. Therefore, we tried to keep the measurement conditions as stable as possible using an interference-free examination room. By evaluating integral changes in SCR, individual variation should be reduced in the current study, but it must still be considered that potential non-responders could distort between group effects [21]. A recent review on physiological abnormalities in patients with ADHD, medications were identified to have effects on autonomic functions. Stimulants were shown to cause an upregulation of the autonomic nervous system that counteract the hypoaroused state. This excitatory effect of ADHD-specific medications has also been demonstrated after discontinuation of the medications [79]. The chosen 24-h washout period should be considered a limitation of the study, as residual effects from previous medications might not have been fully eliminated. Future research should account for the potential for longer-lasting medication effects and implement extended washout periods accordingly. In addition, the clinical profile of ADHD is characterized by a heterogeneity of symptoms, which means that deficits in the functioning of affective processes do not necessarily apply to all individuals with ADHD. For instance, no significant sex differences in emotional competence were identified using the EKF questionnaire, which must also be considered when interpreting the results, as the present sample only partially reflects the impairments of individuals with ADHD that are confirmed in the literature. Moreover, regarding the subdivision of sex, the small sample size must also be taken into account as limitation and can intricate a generalization of the results. In addition, a recruitment bias must always be considered, as young students tend to be more inclined to participate in a scientific and financially remunerated study. This bias in terms of age and sex should therefore be considered as a limitation.

## Conclusion

In summary, the present study was the first to demonstrate sex-dependent effects of affective functioning on undeliberate risky DM behavior in adults with ADHD. The results show that women with ADHD are more likely to engage in risky DM behavior than men with ADHD in the paradigm used and that this behavior might be traced back to alterations in the relation between activity of the autonomic nervous system and intuitive behavior. However, future research should further investigate whether alterations in women with ADHD are related to sensitivity to their own body functions. This insight could then represent a possible approach for additional and sex-specific therapeutic measures. Additionally, the stimulant effect of ADHD-specific medication on autonomic functions should be further explored in the context of DM behaviors. Overall, this study indicates that a greater focus addressing sex-specific deficits is needed in diagnosis and treatment of adults with ADHD.

## Data Availability

The datasets used and analyzed during the present study are available from the corresponding author on reasonable request.

## Abbreviations

ADHD: Attention-deficit/hyperactivity disorder
BART: Balloon analogue risk task
HC: Healthy control
MDD: Major depressive disorder
DM: Decision-making
SCR: Skin conductance response
SMH: Somatic marker hypothesis
BDI: Beck depression inventory
CAARS: Conners adult rating scale
WURS-k: Wender Utah Rating Scale
EKF: Emotionale Kompetenz Fragebogen
DOSPERT: Domain Specific Risk Taking
SPSRQ: Sensitivity to Punishment and Sensitivity to Reward
BSL: Borderline symptom list
aSCR: Anticipatory skin conductance response
rSCR: Reactive skin conductance response
CDA: Continuous decomposition analysis
ANOVA: Analysis of Variance
MANCOVA: Multivariate Analysis of Covariance
RT: Response time
